# Cell–Extracellular Matrix Mechanobiology: Forceful Tools and Emerging Needs for Basic and Translational Research

**DOI:** 10.1021/acs.nanolett.7b04982

**Published:** 2017-12-06

**Authors:** Andrew W. Holle, Jennifer L. Young, Krystyn J. Van Vliet, Roger D. Kamm, Dennis Discher, Paul Janmey, Joachim p. Spatz, Taher Saif

**Affiliations:** † Department of Cellular Biophysics, Max Planck Institute for Medical Research, Jahnstraße 29, 69120 Heidelberg, Germany; ‡ Institute of Physical Chemistry, University of Heidelberg, 69117 Heidelberg, Germany; § BioSystems & Micromechanics IRG, Singapore-MIT Alliance in Research and Technology, Singapore; ║ Department of Biological Engineering, Massachusetts Institute of Technology, Cambridge, Massachusetts 02139, United States; ⊥ Department of Materials Science and Engineering, Massachusetts Institute of Technology, Cambridge, Massachusetts 02139, United States; # Department of Mechanical Engineering, Massachusetts Institute of Technology, Cambridge, Massachusetts 02139, United States; ∇ Molecular & Cell Biophysics Lab, University of Pennsylvania, Philadelphia, Pennsylvania 19104, United States; ○ Institute for Medicine and Engineering, University of Pennsylvania, Philadelphia, Pennsylvania 19104, United States; ◆ Department of Mechanical Sciences and Engineering, University of Illinois at Urbana–Champaign, 1206 West Green Street, Urbana, Illinois 61801, United States

## Abstract

Extracellular biophysical cues have a profound influence on a wide range of cell behaviors, including growth, motility, differentiation, apoptosis, gene expression, adhesion, and signal transduction. Cells not only respond to definitively mechanical cues from the extracellular matrix (ECM) but can also sometimes alter the mechanical properties of the matrix and hence influence subsequent matrix-based cues in both physiological and pathological processes. Interactions between cells and materials in vitro can modify cell phenotype and ECM structure, whether intentionally or inadvertently. Interactions between cell and matrix mechanics in vivo are of particular importance in a wide variety of disorders, including cancer, central nervous system injury, fibrotic diseases, and myocardial infarction. Both the in vitro and in vivo effects of this coupling between mechanics and biology hold important implications for clinical applications.

The idea that physical properties influence biological structure and function has a long history in cell biology and physiology. Classic work by D'Arcy Thompson emphasized the importance of incorporating the laws of physics into biological models.^1^ Many experimental studies and computational models since then have revealed the important effects of cell-generated forces, forces acting upon cells, and physical characteristics of the extracellular matrix on cell morphology and function. A similar understanding of tissue function in vivo remains a challenge for the field, as does adaptation of the revolutionary new tools of molecular biology to biomechanical studies. Nonetheless, the field of mechanobiology, which relates the reciprocity of mechanical and biological interactions, is of increasing interest to many cell biologists as genetics and biochemistry alone are insufficient to explain biological form and function.

## Extracellular Matrix Characteristics Are as Widely Variable as Cellular Responses

**Figure 1:**
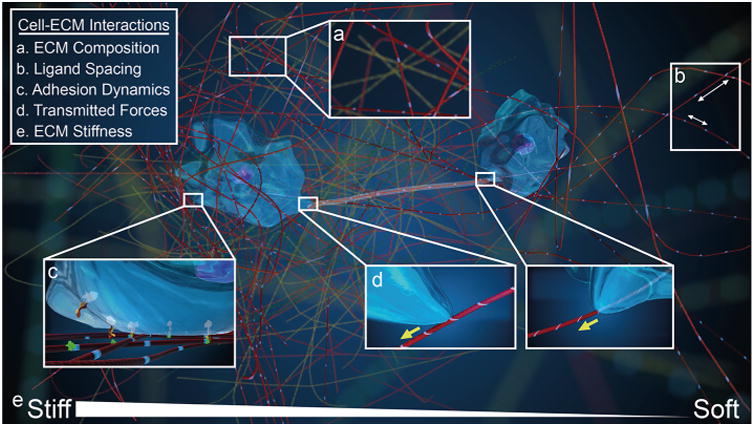
Cell–ECM interactions in a 3D microenvironment. Two cells interact with their matrix microenvironment, illustrating a number of key cell–ECM interactions. (A) Microenvironment composition with different ECM fibers portrayed in yellow and red contributes to mechanical properties of the matrix. (B) The ability of cells to bind specifically to different ECM fibers can result in differential cell ligand spacing in the matrix as a function of fiber density. (C) Cells bind to these ligands via transmembrane integrins, which can be specific to different ECM fiber ligands. (D) As a result of this cell–ECM binding, cells transmit force to the ECM fibers. This tension can be felt by cells at a distance, resulting in mechanical cell–cell communication. (E) ECM fiber density and cross-linking can result in changes in local stiffness. Gradients in this stiffness, as illustrated here, can be features of normal or pathological ECM.

Mechanobiology can be approached from multiple angles. The microenvironment surrounding cells in vivo and in vitro can play a large role in directing cell behavior. Thus, the mechanical aspects of this landscape (i.e., mechanoscape) are important for both understanding cell behavior and building tools designed to replicate it. Most adherent cell types can actively sense the mechanical properties of their surroundings by exerting contractile force, which is transmitted to cell–matrix or cell–cell adhesions. Passive mechanical aspects of the extracellular matrix (ECM) include its bulk and local stiffness and viscoelasticity, ligand density, and topography (Figure 1A,B).^2^ Cells produce and can modify the organization of this ECM, which can vary widely in both composition and cell adhesion characteristics (Figure 1C,D). Thus, these mechanical properties are a direct result of cellular activity, leading to the principle of dynamic reciprocity between the cell and its environment.^3,4^ Conversely, cells can gain mechanical information passively when the ECM exerts a force onto them as tissues are deformed in shear, elongation, or compression, facilitated by static or cyclic mechanical stresses.^5^ Cells can also act upon each other from a distance via traction-induced ECM displacements (Figure 1D).

Cellular responses to these widely variable ECM conditions are equally numerous. Many cell types bind primarily to the ECM, as opposed to binding to other cells. Hence, it is possible to engineer substrates mimicking in vivo mechanical conditions,^6^ place cells on or within them, and observe cell behavior as an output. A tremendous variety of cell outputs have been observed in response to changes in simple substrate stiffness, including cell spreading,^7^ migration,^8–11^ ECM deposition,^12^ stiffness,^13,14^ traction force generation,^15,16^ proliferation,^17,18^ calcium ion concentration,^19^ stem cell lineage commitment^20^ and self-renewal,^21^ cancer cell invasion,^22^ plasticity,^23^ and metastasis,^24^ vascular endothelial sprouting,^25^ and muscle cell phenotype and function. ^26–28^ Mechanisms for these responses are partially worked out and nearly always require actomyosin contractile force generation.^20^ As a more complete understanding of the relationship between cells and their ECM comes into focus, the tools used to sharpen the image will certainly be platforms that combine multiple ECM characteristics and externally imposed strains.^25,29–31^

For other cell types, cell–cell attachments dominate the extracellular landscape. In these environments, mechanotransduction is mediated by various cell–cell junctions, including tight junctions, anchoring junctions, and gap junctions. Cadherins have been found to play a large role in mechanotransduction by linking intercellular adhesions to the cytoskeleton with actomyosin force transmitted through tension-dependent *β-* and *αε*-catenin complexes.^32^ Collective cell movement, which is facilitated by cell–cell attachments, is dependent upon the force sensor Merlin, which prevents local Rac1 activation and allows for polarized migration.^33^ The relative strength of cell–cell interactions can also play a key role in cell migration across biological barriers, such as the extravasation of leukocytes through blood vessels.^34^

## Mechanotransduction Machinery Flows from Integrins and Focal Adhesions to the Cytoskeleton and Ultimately the Nucleus

Cellular responses to the ECM mechanoscape are generally a direct result of mechanotransduction, in which the cell translates mechanical information into a biological response. These responses can occur on both “fast” and “slow” time scales with altered gene expression expected to take longer to develop than simple cytoskeletal or protein alterations.^5^ Cell–ECM mechanotransduction has been shown to occur in different localities from the cell membrane to focal adhesions to the contractile cytoskeleton to the nucleus itself. Ultimately, a complex synergy is required from multiple systems of the cell to properly process and react to extracellular cues.

The first connection between cells and the ECM are integrins, transmembrane proteins that link the interior of a cell with its exterior surroundings. Integrins may function as mechanosensors in some settings.^35^ Force-based conformational changes in integrins have been shown to increase integrin affinity for both ECM proteins and cytoskeletal proteins.^36–38^ In the case of “catch bonds” first observed in rolling leukocytes^39^ but also considered consistent with integrin–ECM binding, an increased tensile force on the bond causes an increase in affinity for the ligand, effectively strengthening the bond. Integrin spacing as a function of ECM ligand presentation also plays an important role in traction force development.^40^

Focal adhesion proteins have been heavily implicated in mechanotransduction, as contractile forces are transmitted from the cytoskeleton to ECM-linked integrins through adhesion-based proteins.^41,42^ These proteins can then differentially change conformation or unfold in response to this force, resulting in the exposure of cryptic binding sites and initiating signaling pathways that ultimately alter gene expression.^43^ Models of force sensitive assemblies of integrins and focal adhesion proteins have predicted stiffness-dependent focal adhesion growth.^44^ Tension on the plasma membrane can also stimulate the force-sensitive opening of ion channels, which can alter integrin conformation and ECM ligand affinity due to both the local changes in pH^45^ and the applied tension.^46^

In the cytoskeleton, myosin-II forces generated on parallel arrays of actin filaments are essential. These cell-generated forces provide, for example, the tension necessary to activate integrin catch bonds and unfold focal adhesion proteins. Thus, the biochemical pathways required to initiate and reinforce cellular traction force generation are key descriptors to mechanotransduction. Although multiple upstream pathways have been implicated in this process, the RhoA/ROCK cascade plays a role in many cell–ECM related cascades, including stem cell differentiation as regulated by the surface area over which cells are allowed to adhere and spread on rigid substrates,^47^ cancer cell invasion and migration,^48^ cell stiffness,^49,50^ and three-dimensional (3D) morphology.^51^ Of course, inhibition of myosin-II also blocks mechanosensitive aspects of these various processes.

For time scales in which gene expression is altered, the mechanotransduction cascade must transmit to the nucleus. Comparison of gene activation in cells plated on substrates of relatively lower and higher stiffness revealed that the transcriptional regulators YAP and TAZ translocate into the nucleus at sufficiently high levels of ECM stiffness via a RhoA-dependent mechanism with cytoskeletal tension promoting nuclear retention.^52^ YAP and TAZ are well-known for their roles in development, growth, and regeneration; therefore a key mechanobiological question is whether YAP and TAZ are triggered by properties such as stiffness in adult tissues independent of proliferation and maintenance of tissue volume. Other studies have shown that applied external strain results in protein kinase translocation to the nucleus within minutes of strain application^25^ and can promote progenitor cell differentiation within hours.^53^ A wide body of work further suggests that the nucleus is itself a mechanosensor and a mechanotransducer.^54–57^ Transmission of force from the periphery to the center of the cell has been modeled to be dependent on local heterogeneities in cellular stiffness, allowing for fast propagation along prestressed cytoskeletal filaments.^58^ Altogether, mechanotransduction of ECM conditions is a complex feedback system integrating multiple cellular processes, locales, and time scales (Supporting Information Video).^59^

## Constitutive Relationships Governing Mechanobiology

The field of mechanobiology draws heavily on its physics and engineering foundations to pursue the development of mathematical models that can predict new phenomena. These models can be broadly divided into three categories: material/matrix characterization, cell–matrix force relationships, and biochemical pathways responsive to mechanical cues.

**Figure 2:**
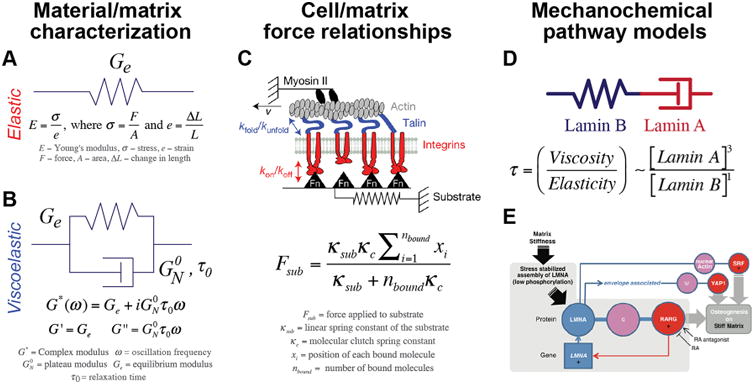
Examples of constitutive relationships defining mechanobiology. Models underlying mechanobiology can include (A) material/matrix characterization, (B) cell–matrix force relationships, or (C) biochemical pathways that are initiated or altered by mechanical cues. Figures adapted from refs 57, 71, and 101.

Mechanical characterization of human tissue and ECM is heavily influenced by materials science principles. Many investigations into cell–matrix mechanical interactions idealize the substrate materials to be described as linear solids,^10,20,55^ which can be modeled as a spring or described with a time-invariant elastic constant expressed as Young's elastic modulus (E) or shear modulus (G) (Figure 2A). As such, substrate stiffnesses are often reported in units of Pascal (Pa), or force per unit area, with the physiological range spanning from 100 Pa in neural tissue to 10 kPa in muscle tissue to over 1 GPa in mineralized bone.^60–64^ However, multiple biological materials and engineered polymers or gels can be described more accurately as viscoelastic,^65–67^ in which both time-dependent viscous and time-independent elastic components contribute significantly to rates and extents of deformation. When describing the characteristics of linear viscoelastic substrates, a shear storage modulus (*G′*) representing the spring-like elastic component and a shear loss modulus (*G″*) representing the dashpot-like viscous component can be reported. The classical Kelvin–Voigt model of a linear viscoelastic solid utilizes a spring in parallel with a dashpot (Figure 2B). Viscoelastic hydrogel substrates have been used to show that with a constant *G′* of 4.7 kPa, myogenic differentiation of stem cells is maximized by substrates with a *G″* of 130 Pa compared to those with a *G″* of 1 Pa.^68^ In either case, the gels were considered solids and not liquids, because *G′* ≫ *G″*, but the deformation time scale of the gels differed and it is inferred that this in turn modulated cell forces and response times.

To build constitutive relationships between cells and the ECM to which they are attached, the models must become more complex, as the force generated by cells must pass through integrin/focal adhesion linkages to the material itself, with some proteins acting as molecular clutches. To evaluate the mechanical clutch theory of cell migration and mechanotransduction, initial approaches modeled the combined mechanics of retrograde actin flow and substrate stiffness along with binding kinetics of integrins.^69^ Later work refined this model by accounting for differential binding dynamics of various integrin-ECM protein interactions.^70^ Most recently, the inclusion of focal adhesion protein dynamics has resulted in a model that is in better agreement with experimental results (Figure 2C).^71,72^ Future models will likely build on this stepwise progress by incorporating more complex downstream events like cytoplasmic protein translocation and gene activation.^73^

Finally, mirroring the development of mathematical models to describe biochemical reactions (e.g., enzyme kinetics, tissue growth, solute diffusion), a wide variety of constitutive relationships underlying mechanochemical outcomes have been proposed. These models, which aim to connect extracellular mechanical properties with protein activity in the cell and subsequent gene activation provide valuable insights into control parameters of cell behavior. For example, the mechanoresponsive nuclear intermediate filaments Lamin A and Lamin B have been shown to follow equations governing distinct aspects of polymer physics.^57^ Nuclear viscosity scales to the third power of Lamin A concentration, a protein exhibiting some intracellular mobility,^74^ while nuclear stiffness (i.e., elastic component of viscoelastic deformation) scales linearly with Lamin B concentration. Accordingly, the time *τ* needed for viscous Lamin A dissipation of energy stored in the elastic Lamin B network follows the relationship in Figure 2D.

These mechanical relationships reflect the dynamics of structural proteins that in turn regulate nuclear entry of transcription in a feedback-based gene expression circuit, connecting the dots from matrix properties to cellular mechanics to gene expression (Figure 2E).^53,57,75^ Ultimately, multiscale models governing the interactions between cells and their mechanical microenvironment as well as the resulting phenotypic changes have great potential to shape future basic research as well as provide a basis for translational applications.

## Grand Challenges Facing the Field of Mechanobiology

### Deeper Characterization of the Physical, Chemical, and Cellular Microenvironments of Pathophysiological Cell Niches

In keeping with efforts to establish the most basic fundamentals of cell biology, one major goal must be to establish generalities of different tissues in terms of materials properties. This includes efforts to understand ECM remodeling dynamics during homeostasis and pathological conditions, the relation between ECM remodeling and mechanical changes, and the correlation of cell phenotypes with their past and current microenvironments. To determine how cells function correctly and find causes for why they malfunction, systems mimicking normal and pathological ECM must be developed and improved. A comprehensive characterization of in vivo forces and mechanical landscapes has not yet been achieved; this will be necessary in order to design and implement in vitro ECM tools that perfectly mimic in vivo counterparts. During this process, multiple size scales on which mechanobiology is relevant must be united, from proteins to cells to tissues to organisms. Heterogeneity among cells within a given cell population is also an important component of any given matrix niche, and thus a better understanding of the role of cell–cell interactions in these mechanical processes will be necessary. Once full characterization of the mechanical microenvironment is realized, manipulating that environment to control cell behavior can become a functional, clinically relevant goal.

### Systems-Level Understanding of Pathways That Underlie Mechanosensitive Responses

In parallel with advances in characterizing the extracellular landscape, the role of intra-cellular signaling in response to the mechanoscape should be explored. The question of how cells transduce biomechanical cues into biochemical cascades, which can then also elicit further biomechanical responses, must continue to be answered. From a physical perspective, the degree of mechanical coupling between different elements of the cytoskeleton remains unclear. From a biochemical perspective, the signaling pathways that power mechanotransduction should also be further elucidated.

As mechanotransduction often drives changes in gene expression, this will include a complete accounting of the many factors that enter or leave the nucleus in response to mechanical signals. Understanding of changes in chromatin folding and cellular epigenetics and their intersection with cell mechanics will also prove fruitful. Information on how far upstream mechanical signaling can be replicated or affected will allow for the development of drugs capable of nonmechanically stimulating mechanical pathways.

### Mechanobiology Toolbox: Development and Standardization of the Models and Tools Used To Understand Cell–ECM Interactions

New fields must blaze new pathways with respect to models, techniques, and tools, leading to the invention, testing, and improvement of methods. However, for sufficient maturation these methods must be agreed upon, standardized, and adopted in order to reduce inconsistency in experimental observations. Consensus is needed for standard cell and tissue selection, substrate material fabrication and characterization, and the mechanical frameworks and mathematical models used to characterize the mechanoscape.

In addition, new methods must be developed to address areas in which current methods are lacking. Accurate and noninvasive measurements of local cell and matrix mechanical properties in situ are needed both to fully characterize the mechanoscape, which can vary widely in response to physical or biological stimuli,^76^ and to properly inform attempts to replicate in vivo environments in vitro. A recent first step in that direction is a multilab project comparing a wide range of methods to measure the elastic modulus of a single cell under standardized methods.^77^ High throughput, biomimetic 3D culture systems compatible with improved microscopy techniques to monitor the responses of cells and tissues to mechanical perturbations will allow for a better understanding of the coevolution of cells and the matrix, as was recently demonstrated in a system capable of both high content imaging and controlled applied strain.^25^ Robust in silico systems utilizing proteomics data and mechanical simulation to predict force-induced conformational changes of proteins could replace costly and time-intensive deformation experiments using atomic force microscopes, laser tweezers, and Förster resonance energy transfer sensors.

In conjunction with the development of these new tools, the establishment and implementation of international online databases of methods, models, and protocols will be key for driving consensus within the field. One ongoing forum for this dialogue is the online wiki MBInfo, produced by the Mechanobiology Institute of the National University of Singapore, with stated goals of defining and standardizing mechanobiology. As development and standardization can at times be at odds, online spaces of this type can serve as a sounding board, connecting the international mechanobiology community and leading to greater research efficiency.

## Entering the Clinic: Translational Impacts of Mechanobiology

The role of mechanics in biology has been demonstrated and emphasized in basic science laboratories within both the biology and engineering communities. Incorporating mechanobiology principles into new or existing clinical treatments is an important next step which will require both new collaborations and new applied research efforts. Similar to the accepted clinical concept of biocompatibility, mechanocompatibility, and ECM composition must be taken into account when designing and implementing medical implants, therapeutic interventions, or cell-based therapies. While mechanobiology principles can be applied to all diseases, including disorders as varied as glaucoma, muscular dystrophy, progeria,^78^ and multiple sclerosis,^53^ three areas that have received an increasing amount of attention and collaboration are highlighted here.

### Cancer

**Figure 3:**
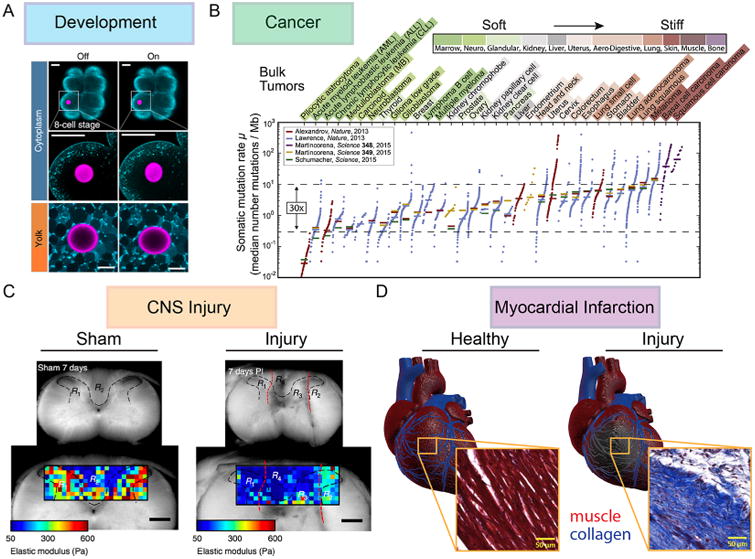
Unique cell–matrix microenvironments. (A) In the developing embryo, stiffness gradients begin to appear as early as the blastula phase. Using ferrofluid microdroplets as mechanical actuators, Serwane et al. showed that droplet deformation under identical magnetic fields yields more deformation in the cytoplasm of a blastomere than in the yolk, indicating a stiffer yolk. These droplets can be actuated dynamically during the entire course of embryo development to measure viscoelastic properties of embryonic tissues.^102^ (B) Pfeifer et al. recently investigated the cancer cell–ECM microenvironment by finding a correlation between the stiffness of the tissue surrounding a tumor and the somatic mutation rate within the tumor.^80^ This has been hypothesized to be the result of increased ECM deposition in stiffer tissues requiring migrating cancer cells to contort their nuclei, causing a depletion of DNA repair factor and a subsequent increase in DNA damage.^103^ (C) Clinical translation of mechanobiology research to the field of CNS regeneration is an urgent need. Atomic force microscopy analysis of both uninjured regions and stab injury sites of the neocortex performed in Moeendarbary et al. revealed that brain tissue softens after injury, and that this softening extends to regions nearly half a millimeter away from the injury and persists for over 3 weeks.^104^ (D) Another potential clinical application for mechanobiology principles is in myocardial infarction, where cell death in the infarct zone leads to increased matrix deposition and stiffening. This ECM alteration results in decreased cardiac output for post-MI patients.^105^ Images adapted from refs 80, 102, 104, and 105.

Cell–ECM interactions in the cancer microenvironment present a promising avenue for clinical investigation,^79^ especially because somatic mutation rate scales with stiffness of the normal tissue (Figure 3B).^80^ ECM-targeted drugs aim to either inhibit specific matrix interactions that contribute to ECM-conferred chemoresistance, or to alter the tumor microenvironment such that cell behavior or drug delivery can be better controlled. ECM production within the tumor is upregulated, resulting in most cases in enhanced stiffness compared to healthy tissue.^81^ This higher matrix stiffness, correlated with more densely packed ECM fibers, presents two problems: first, increased stiffness can promote metastatic behavior in cancer cells,^82^ and second, delivery of drugs and perhaps immune cells throughout the entirety of the tumor is hindered.^83^ TGF-*β* inhibitors, for example, reduce the secretion of ECM proteins^84^ in order to prevent further ECM alterations. As tumor cell metastasis is the major cause of death, several drugs have been developed to prevent the migration of metastatic cells.^85^ These metastatic cells work their way through the body by degrading ECM via production of matrix metalloproteinases (MMPs) or other matrix-reducing enzymes (e.g., heparanase). Many therapies aim to inhibit the production of such enzymes in order to prevent extravasation of invasive cells.^86,87^ Immuno-oncology^88^ is a rapidly developing area of translation with the emergence of new and clinically effective molecules (e.g., checkpoint inhibitors) and engineered cells (e.g., cancer antigen receptor T-cells, or CARTs). T-cell activation has been shown to be sensitive to nanoscale antigen spacing in the ECM,^89,90^ echoing basic mechanobiology observations about ligand presentation. However, successful deployment of these reagents against solid tumors^91^ remains elusive and generally relies on infiltration of immune cells that encounter the physical barriers cited above.

### CNS Injury

Central nervous system (CNS) injury remains a key area of focus for clinical tissue engineering (Figure 3C). Accordingly, the role of cellular mechanotransduction in both healthy and injured CNS tissue is coming into focus. As mechanical forces are the direct cause of traumatic brain injury, it stands to reason that mechanics on a cellular scale play a major role in the biological response. These forces cause deformation of mechanically heterogeneous neural tissue with localized forces at the point of impact and generalized forces throughout the skull resulting from both inertia and pressure waves.^92^ Pressure waves are likely to result in increased force across integrins, activating the Rho pathway and thus stimulating cellular contractility, leading to further axonal injury. Preclinical tests of ROCK inhibitors applied in response to injurious forces confirmed that a reduction in cellular contractility can reduce the incidence of axonal injury.^93^ Transient membrane tearing and axonal swelling are also common responses to cellular strain that likely lead to an ion-induced upregulation of proteases promoting apoptosis. Several drug candidates have been explored that promote membrane resealing post-trauma^94,95^ but have not yet reached clinical trials.

Another key feature of many CNS injury types is demyelination of the lesion. Myelin wraps around the axons of healthy neurons, and is required for both neuron health and efficient signal transduction. Through processes that are incompletely understood, myelin sheaths are destroyed and are not repaired efficiently in response to mechanical trauma such as TBI, and under chronic diseases including multiple sclerosis. This protein-rich wrapping is produced by a CNS glial cell type termed oligodendrocytes, and these cells are mechanosensitive to local stiffness^53,96^ and to applied strain through the same RhoA/ROCK pathways shared by many other cell types.^53^ Mechanobiology of glial cells including oligodendrocytes is recognized increasingly as an important target for development of clinical remyelination strategies. In this context, drug development will be aided by in vitro platforms that exhibit mechanical and structural features of CNS tissue, as those cues can affect cells' response to drugs in the in vitro experiments that are used to select compounds for in vivo clinical trials.

### Myocardial Infarction

Regenerative medicine is increasingly informed by mechanobiology investigations into embryo-genesis and development (Figure 3A). Following myocardial infarction (MI), survival is greatly determined by post-MI complications, including infarct rupture, heart function repression, and progression to full heart failure. These complications are often directly or indirectly tied to the regeneration process, in which the infarcted tissue initially becomes softer and thinner due to cell death. Over time the region is remodeled and replaced by a scar that can be more than 3-fold stiffer than the surrounding healthy tissue^97^ (Figure 3D). This stiffening has been found to alter cardiomyocyte beating frequencies and percentages.^98^ As such, one proposed clinical strategy has been to modify infarct mechanical properties to reduce cardiac remodeling and mitigate improper mechanosensitive signaling. The administration of soft tissue filler, causing an increase in early infarct stiffness and a reduction in infarct expansion and remodeling in animal models.^99^ However, other studies have shown that more compliant infarct regions are key to reducing remodeling over a period of 8 weeks,^97^ suggesting that future clinical strategies for infarct maintenance must take the dynamic relationship between stiffness, remodeling, and cardiac function into account.

When regenerative medicine includes use of mesenchymal stem cells to repair MI, such as direct administration of cell-based therapies to the injured sites, in vitro mechanics also play a role in translational studies. It is recognized increasingly that stem cells expanded in vitro are heterogeneous populations of mesenchymal stromal cells, and that the cell culture environment can foster population heterogeneity upon successive passages on standard substrate materials.^100^ Those materials such as polystyrene (*E* ∼ 10^9^ Pa) are orders of magnitude stiffer than the tissues from which the stem cells originated (*E* ∼ 10^2^–10^6^ Pa) or are targeted for therapeutic delivery. As with many other cell types, mesenchymal stromal or stem cells are mechanosensitive to such cues,^20,41^ and thus translation of such cell-based regenerative medicine strategies will be advanced by substrates that better replicate in vivo environments and by in vitro protocols that mitigate cell population heterogeneity in the cell therapy products.

## Conclusions

The maturing field of mechanobiology is by definition interdisciplinary, combining multiple fields of biology with physics, mechanics, materials science, and thermodynamics. As a result, it is unsurprising that the pioneers of this field specialized in basic science; the generation of testable concepts and model systems from other disciplines have been and will continue to be valuable. In pursuit of the grand challenges confronting mechanobiology, a number of new experimental, theoretical, and computational platforms and tools must emerge. Clinical results can inform and benefit from the development of these platforms. Thus, opening the field up to substantive collaborations with oncologists, clinicians, and other medical experts will result in a productive exchange of ideas in both directions, providing new observations for the pursuit of basic research and allowing for clinical interventions to be viewed through the lens of substrate mechanics and cell—ECM interactions.

## Supplementary Material

movie Mechanotransduction of ECM conditions showing a complex feedback system integrating multiple cellular processes, locales, and time scales (MP4)
